# Importance of epidemiological surveillance of leprosy: analysis of the occurrence of leprosy in intra-domiciliary contacts in a capital in the Brazilian northeast region

**DOI:** 10.1590/0037-8682-0507-2019

**Published:** 2020-03-16

**Authors:** Luciana Cavalcante Trindade, Lourdes Conceição Martins, Danielle Medeiros Marques, Micheline da Silveira Mendes, Fernando Luiz Affonso Fonseca, Luiz Alberto Amador Pereira

**Affiliations:** 1 Universidade Católica de Santos, Programa de Pós-Graduação Stricto Sensu em Saúde Coletiva, Santos, SP, Brasil.; 2 Faculdade de Medicina do ABC, Programa de Pós-Graduação Stricto Sensu em Ciências da Saúde, Santo André, SP, Brasil.; 3 Universidade Federal da Paraíba, Departamento de Promoção da Saúde, João Pessoa, PB, Brasil.; 4 Fundação Osvaldo Cruz, Programa de Pós-Graduação Stricto Sensu em Saúde Pública, Recife, PE, Brasil.

**Keywords:** Leprosy, Epidemiological surveillance, Neglected diseases, BCG vaccine

## Abstract

**INTRODUCTION:**

Intra-domiciliary contacts are a group with the highest risk of developing leprosy.

**METHODS:**

A cross-sectional study of intra-domiciliary contacts of new leprosy cases was conducted. A descriptive analysis of the variables was performed.

**RESULTS:**

Among 190 contacts, 63% were invited to visit the health unit, and 54.2% received the BCG vaccine. The prevalence of leprosy among the contacts was 4.7%.

**CONCLUSIONS:**

The occurrence of leprosy among the contacts was high and similar to that found previously. There were failures in surveillance actions carried out by health units. Never-before treated cases were found.

The intra-domiciliary contacts of patients with multibacillary leprosy are more likely to develop the disease than the general population^1^. Epidemiological surveillance, including dermatologic evaluation and vaccination of contacts with BCG, has been reported by the Ministry of Health of Brazil as a primary action for the control of leprosy^2^.

In Brazil, the examination of intra-domiciliary contacts, part of the *Priority Actions of the Program of Health Surveillance Actions,* serves as a basis for the construction of the operational indicator which is related to the examination of contacts. The operational indicator has presented unfavorable results in Brazil over the years, with gradual improvement only since 2012^3^ ^,^ ^4^. The situation with regard to this indicator is even worse in Paraíba (PB), a Northeastern Brazilian state, which had precarious to regular contact coverage in recent years, reaching 60.35% in 2017 and remaining the second or third worst ranked in this indicator^3^. In João Pessoa, the state capital, the coverage of contacts was 52.6% in 2017, which is considered regular^4^. 

There are no current studies on the prevalence of leprosy cases, and no studies on the relationship between intra-domiciliary contacts in Paraíba and the role of epidemiological surveillance have been conducted. Thus, the objective of this cross-sectional study was to evaluate the occurrence of leprosy cases in intra-domiciliary contacts of diagnosed patients and to evaluate the execution of surveillance actions on intra-domiciliary contacts by health units in João Pessoa, the municipality that has had the highest number of cases reported in the state over consecutive years^5^. 

The study population was intra-domiciliary contacts of new leprosy cases living in João Pessoa/PB who were notified between January 1 and December 31, 2012, referred to as “index cases” in this study. For the purpose of this research, an intra-domiciliary contact is every person who cohabited with the index case at the time of diagnosis. The index cases and the corresponding contacts were identified and located using information of the state database of SINAN (Notification of Invalidity Information System) and medical records of the State Reference Service. The collection of data occurred from April to July 2014.

Nurses visited the contacts at home. Patients underwent a physical examination and filled out the survey form with the variables of interest. Individuals with cutaneous lesions were evaluated by dermatologists.

The surveillance actions developed by the health units were evaluated using the responses of contacts to three questions: whether they were invited to visit the health unit (HU), whether they visited the HU, and whether they had received counseling on the BCG vaccine. 

Cases of leprosy in the contacts - even those who had already been treated or were under treatment - were counted when calculating the prevalence of the disease among this population. For such cases, the clinical form (according to the Madrid classification), the operational classification (according to the classification of the World Health Organization, WHO), the therapy, and the disability grade were recorded. 

A descriptive analysis of all study variables was performed in terms of their absolute and relative values. The software *Statistical Package of Social Science for Windows*, version 17, was used for data analysis. All ethical aspects were complied with. 

In 2012, 94 new cases of leprosy were diagnosed in the city of João Pessoa/PB, along with 283 contacts. One (0.01%) case was excluded because it was reported erroneously, and 21 (22.3%) cases could not be located. Thus, 72 index cases and 190 related contacts were included in the study. 

Most index cases were male (58.4%), 15 years or older (66%). The mean age was 40 years (SD = 17.7). The most frequent clinical form was tuberculoid (34.7%), followed by borderline (27.8%), and the operational classification was multibacillary (56.9%). The majority presented zero disability grade diagnosis (56.9%) and received the diagnosis at the reference hospital (84.7%).

Most of the contacts (61.1%) were female, single (59.5%), and white (52.6%), with a family income of one to five minimum wages (65.8%). The most common education level was between eight and 11 years (35.3%). The mean age was 45 years old. Most denied smoking (85.3%) or using alcohol (62.6%), , illicit drugs (94.7%), and medication (66.8%). In relation to parentage, the most frequent link between the contact and the index case was son (27.9%), followed by spouse (21.0%). As for profession, the most common was student (33.7%), followed by homemaker (21.6%). 

Regarding surveillance actions, 120 (63.2%) contacts reported that they were invited to visit the health unit for evaluation, 99 (52.1%) visited it, and 103 (54.2%) said they were advised to receive the BCG vaccine.

Sixteen (8.4%) contacts had lesions suggestive of leprosy, of which three (18.7%) had already received the diagnosis of leprosy and were under treatment. The diagnosis was discarded in 11 (68.8%), and two (12.5%) were not evaluated. Thus, never-before treated cases were diagnosed among the contacts. 

Eight (4.2%) intra-domiciliary contacts reported being treated for or having undergone treatment for leprosy. Table 1 shows socioeconomic, demographic, and clinical information about them. The mean age was 49.6 (SD = 18.3) years. Most were male, single, and white. Regarding the clinical and operational characteristics of index cases, the most frequent clinical classification was borderline, the most common operational classification was multibacillary, and the most common disability grade at diagnosis was ‘1’. Seven (87.5%) people were diagnosed in the reference hospital. Half of them had no BCG scars, and the rest had one scar.

**TABLE 1:** Distribution of the number and percentage of contacts with treated leprosy according to socioeconomic and demographic variables and clinical and operational characteristics. João Pessoa, 2012

	N	Percentage
**Gender**		
Male	6	75.0
Female	2	25.0
Total	8	100.0
**Age**		
20-30 years	2	25.0
40-50 years	3	37.5
Over 60 years	3	37.5
Total	8	100.0
**Marital status**		
Single	4	50.0
Married	2	25.0
Stable union	2	25.0
Total	8	100.0
**Ethnicity**		
White	6	75.0
Brown	2	25.0
Total	8	100.0
**Education level (years)**		
No schooling	1	12.5
4-7	3	37.5
8-11	3	37.5
12 years or more	1	12.5
Total	8	100.0
**Use of alcoholic beverages**		
Yes	4	50.0
No	4	50.0
Total	8	100.0
**Use of medication**		
Yes	3	40.0
No	5	60.0
Total	8	100.0
**Clinical form**		
Tuberculoid	1	12.5
Dimorph	5	62.5
Virchowian	1	12.5
No information	1	12.5
Total	8	100.0
**Operational form**		
Paucibacillary	1	12.5
Multibacillary	7	87.5
Total	8	100.0
**Disability grade**		
Zero	1	12.5
One	4	50.0
Two	1	12.5
Not evaluated	1	12.5
No information	1	12.5
Total	8	100.0

One of the families had two index cases, both diagnosed in 2012 (co-prevalence). Only for the calculation of prevalence, the case that received a later diagnosis was counted as *contact with leprosy*. Thus, nine study contacts were diagnosed, with a prevalence of 9/191 contacts (4.7%).

Information from the contacts diagnosed with leprosy relating them to the corresponding index cases is presented in Table 2. The operational form of the contact and the corresponding index case was coincident in four (44.4%) situations; in other situations, the contact was diagnosed with a multibacillary form, whereas the index case had a paucibacillary form. Regarding the year of diagnosis, five (62.5%) contacts were diagnosed before the index case. Three (33.3%) contacts were children of index cases, three (33.3%) were spouses, one (11.1%) was a sibling, one (11.1%) was a son-in-law, and one (11.1%) was a brother-in-law.

**TABLE 2:** Clinical and operational form and disability grade of the index case and the corresponding contact, and degree of kinship between them. João Pessoa, 2012

Clinical form of the	Clinical form of the	Operational form of	Operational form of	Year of contact	DG
index case	contact	the index case	the contact	diagnosis	index case
D	B	MB	MB	2005	0
T	L	PB	MB	2007	0
T	B	PB	MB	2012	1
T	B	PB	MB	2010	1
T	T	PB	PB	2010	0
B	NI	MB	MB	2012	1
T	B	PB	MB	2013	0
T	B	PB	MB	2013	0
T	T	PB	PB	2012	1

*Including a co-prevalent case as contact; **IC:** index case; **B:** borderline; **T:** tuberculoid; **L:** lepromatous; **NI:** no information; **DG:** disability grade; **PB:** paucibacillary; **MB:** multibacillary.

The increased risk of contacts developing leprosy in relation to the general population has been described in Brazil since the 1940s and has been corroborated by several studies over the years^6^ ^,^ ^7^. In the present study, 4.7% of the contacts were affected by the disease. There was an increased prevalence of leprosy cases in intra-domiciliary contacts when compared with the general population, which was 1.40% in Paraíba in 2012^8^. The prevalence was higher than that found by Düppree et al*.* ^6^, Wambier et al*.* ^7^, and Araújo et al*.* ^9^ and lower than that reported by other authors^10^ ^,^ ^11^. 

Half of the contacts in the present study who developed the disease were diagnosed before the index case, suggesting that they constituted the true primary cases in the domiciliary epidemiological chain. These data were corroborated in situations in which the contact presented a multibacillary form, while the respective index case was paucibacillary, as the multibacillary patient is considered the main source of infection of the disease^3^. 

Four index cases of our study were diagnosed two to seven years after the respective contact. If we consider the intra-domiciliary contact of the research as the primary source of infection for them, there may have been a failure in the health network in detecting the disease early or not having guided these individuals on the signs of the disease. However, the design of the study did not allow for knowing whether the so-called index cases were evaluated and received guidance regarding the development of the disease and BCG vaccination when they were still intra-domiciliary contacts. The fact that all patients had a polarized clinical form of the disease and little more than half showed a disability grade ‘1’ at diagnosis suggests that, in fact, a late diagnosis was made.

In our study, more than 85% of the index cases and 87.5% of the contacts received the diagnosis at the reference service. This reflects difficulties with the decentralization of actions related to leprosy, which may indicate a failure in primary care to diagnose the disease in a timely manner by encouraging the occurrence of disability and sequelae and by perpetuating the chain of transmission of the disease^12^.

Regarding contact surveillance actions by the health units, a high percentage of contacts reported not being invited to visit the health unit, and only slightly more than half reported that they visited it and were instructed to take the BCG vaccine, which could have indicated a failure of health services in these important actions, similar to what was perceived by Romanholo et al*.* ^13^ in a cross-sectional study carried out in Rondônia. 

Despite the deficiencies in the health system, negligence by the individuals regarding their own health is also possible. In this regard, Dessunti et al*.* commented that the actions of contact surveillance by the health units are not effective, and contacts do not visit health units even when requested^14^. 

Helena et al*.*, in a qualitative study developed to evaluate the health professionals' perceptions about contact surveillance, concluded that professionals highlight difficulties in the implementation of this strategy related to users and work overload, despite recognizing the importance of this strategy^12^. 

Following the considerations of Dessunti et al*.* ^14^ and Helena et al*.* ^12^ on the role of basic health care, as well as considering the centralization of the diagnosis of cases in our study, adopting additional education and awareness strategies with intra-domiciliary contacts may be necessary. 

The limitations of the study relate to its design, since the demographic information was secondary and collected early, which leads to losses due to inconsistencies and changes of residence. In this regard, the Ministry of Health recognizes, among leprosy surveillance strategies, the need for updating of patients' addresses and their contacts^15^.

Furthermore, as this is a cross-sectional study and leprosy is a disease with a long incubation period, the subclinical cases that supposedly exist among contacts would not have evolved. Regarding this, in the most recent Technical-Operational Manual of the Ministry of Health on leprosy, the recommendation for contacts is to maintain surveillance for a period of five years, a strategy that is adequate when considering the characteristics of the disease^15^.

In conclusion, the occurrence of leprosy among the study contacts was high and similar to that found by other authors. There were failures in surveillance actions carried out by health units. The contact control measures established by the Ministry of Health, in addition to strategies compatible with local variations of endemicity and education of the population, may provide adequate monitoring of contacts, improving the detection and prevention of disabilities and reducing stigma related to a late diagnosis of the disease. 
